# Coverage Path Planning Methods Focusing on Energy Efficient and Cooperative Strategies for Unmanned Aerial Vehicles

**DOI:** 10.3390/s22031235

**Published:** 2022-02-06

**Authors:** Georgios Fevgas, Thomas Lagkas, Vasileios Argyriou, Panagiotis Sarigiannidis

**Affiliations:** 1 Department of Computer Science, International Hellenic University, 65404 Kavala, Greece; gefevga@cs.ihu.gr (G.F.); tlagkas@cs.ihu.gr (T.L.); 2 Department of Networks and Digital Media, Kingston University, Surrey KT1 2EE, UK; 3 Department of Informatics and Telecommunication Engineering, University of Western Macedonia, 50100 Kozani, Greece; psarigiannidis@uowm.gr

**Keywords:** coverage path planning, unmanned aerial vehicle, cell decomposition, decomposition methods, energy-aware approaches, energy optimal path, multi-robot systems, multi-UAV

## Abstract

The coverage path planning (CPP) algorithms aim to cover the total area of interest with minimum overlapping. The goal of the CPP algorithms is to minimize the total covering path and execution time. Significant research has been done in robotics, particularly for multi-unmanned unmanned aerial vehicles (UAVs) cooperation and energy efficiency in CPP problems. This paper presents a review of the early-stage CPP methods in the robotics field. Furthermore, we discuss multi-UAV CPP strategies and focus on energy-saving CPP algorithms. Likewise, we aim to present a comparison between energy efficient CPP algorithms and directions for future research.

## 1. Introduction

In recent years, due to rapid technological development, UAVs and sensors they can carry have been developed to the extent that they can cover a wide range of applications [1] that cannot be satisfied by other types of robots [2]. Some of the applications are precision agriculture [3,4], search and rescue [5], firefighting [6], law enforcement [7], powerline inspection [8], oil and gas [9], disaster management [10], and cell network expansion [11]. However, a fundamental problem is the optimal use of autonomous aircraft in terms of time and space [2].

Recently, CPP algorithms have been developed, considering the parameters required for more efficient data retrieval from remote sensing sensors [12,13]. In addition, algorithms have been developed that use multi-UAV to cover the area, thus reducing the coverage time of the area of interest [14,15]. The way to cover an area with autonomous robots differs depending on the algorithm [2,16,17]. In the literature, CPP algorithms use different methods (e.g., grids, graphs, and neural networks) with calculations performed online or offline for known or unknown areas [18].

The CPP algorithms can be classified into two main categories: offline and online [19]. Offline algorithms need to know the environment and the information included, such as obstacles and the geometry of the area of interest. Of course, in a real-life environment, many dynamic parameters cannot be known in advance. Offline algorithms have prior knowledge of the coverage area environment [20]. They also provide more efficient and convenient route plans and use less central processing unit (CPU) power than online algorithms [21].

The online algorithms are based on real-time environment data retrieved from onboard sensors to cover the area of interest. Online algorithms do not fully understand the coverage area environment, and the coverage path is executed in real-time by the UAV after processing the data using the sensors it carries. The benefits of online algorithms are the design of the in-flight route to complete the mission regardless of unforeseen situations and the unnecessary prior detailed knowledge of the coverage area [20,22].

Furthermore, there are two categories of problems in area coverage: single coverage and repeat coverage. The goal of single coverage is to cover the entire area of interest and, at the same time, minimize the time and distance traveled by the coverage route [23]. On the other hand, repetitive coverage aims to repeatedly cover all points of interest in the area, maximize the frequency of visits to points of interest, and minimize time and total coverage [24].

This paper aims to present the CPP methods and approaches used by UAVs, focusing on energy-saving CPP methods, such as using the direction of the wind in the cover area [25]. The CPP problem is the optimal motion of the robot in a specific area that includes obstacles to cover this area with minimum overlapping and the shortest path. In the case of a UAV in a three-dimensional area, the shortest path is related to the sensor’s footprint. Of course, as the altitude of flight is higher, the footprint is more extensive, which means the shortest path. On the other hand, the higher the flight altitude of the UAV, the bigger the ground sample distance (GSD) and the lower the image quality. GSD is the distance between pixel centers measured in the ground. However, there are a lot of other limitations, such as no-flight zones, that must be computed during path planning to avoid obstacles [26].

Many surveys present studies related to UAV trajectory planning in an environment with obstacles [27], UAV autonomous guidance [28], and in specific applications, such as remote sensing with UAVs in precision agriculture [29]. A survey on CPP methods for mobile robots was presented by Choset, who classified the approaches in two classes [19]. The robots follow simple rules, but the success of area coverage is not guaranteed to be classified as a heuristic approach. On the other hand, the complete methods using cellular decomposition guarantee coverage. Moreover, the author mentions the flight time, which can be minimized by using multiple robots and reducing the number of turns.

The most recent surveys regarding the CPP methods for robotics or UAVs are presented in Table 1. Cabreira et al. [30] present a survey of the decomposition methods, UAV and Multi-UAV CPP methods, and energy-saving algorithms. Galceran and Carreras [20] present a survey of the decomposition methods and ground multi-robot strategies. Additionally, Almandhoun et al. [31] present Multi-UAV CPP methods in their survey. Chen et al. [32] present a survey of CPP methods using UAV or Multi-UAV. The existing surveys of CPP methods considering unmanned ground vehicles (UGV) and the surveys of CPP methods using UAVs extend the UGV’s CPP methods. However, many factors, such as the sensors’ weight, the flight endurance, direction, and intensity of the wind, must be considered when using UAVs on CPP methods developed for UGVs.

Table 1 compares the present work to already existing surveys of CPP methods for robotics or UAVs. The present paper is focused not only on surveying the CPP methods for UAVs, but also on: (a) examining all the decomposition methods, (b) reviewing the multi-robot strategies, (c) the multi-UAV’s and standalone UAV’s CPP methods, (d) UAVs’ energy-saving CPP algorithms, and (e) the comparison of the energy-saving CPP methods. Our approach proves to be the most complete regarding the variables considered for the survey comparison.

The key contributions of this work can be summarized as follows:

- A review of the decomposition methods in different shapes of the area of interest, such as rectangular, concave, irregular, and convex polygons, has been presented.
- A presentation of multi-robot and multi-UAV CPP strategies based on single robot approaches, methods that guarantee the mission’s completeness, and bio-inspired methods that perform coverage under uncertainty.
- A review of energy-saving algorithms and the limitations of them, according to the UAV’s constraints and environmental conditions.
- A discussion of the CPP methods’ limitations, how to overcome them, and directions for future research on energy-saving CPP algorithms.

This paper is organized considering the CPP methods, multi-UAV strategies, and energy-saving algorithms. Section 2 focuses on a detailed analysis of the systematic review research methodology. Section 3 reviews all decomposition algorithms, multi-robot CPP strategies, multi-UAV CPP methods, and presents UAVs’ energy-saving CPP algorithms and a comparative table. Finally, directions for future research on energy-saving CPP algorithms are given in Section 4.

Our review considers the research gap concerning the differences between UGV CPP methods and the UAV CPP methods. Furthermore, our review presents the limitations of the UAVs considering environmental conditions, such as the intensity and direction of the wind. A detailed discussion about the main aspects of multi-robot and multi-UAV CPP methods is also provided. Our review focuses on approaches related to UAV energy-saving algorithms and a discussion of the combination of these algorithms considered for future research.

This paper aims to inform the reader of the coverage path planning approaches in different shapes of the area of interest, including rectangular, concave, and polygons, according to the decomposition method employed. Furthermore, we explore the limitations of the CPP methods between UGVs and UAVs, the latest multi-robot and multi-UAV CPP strategies, and the energy-efficient algorithms for UAVs. Finally, our review considers the performance metrics and the limitations of these methods.

## 2. Methods

For the present work, a systematic review research methodology was adopted. In that context, a range of platforms was sourced for information. Most of the sources cited in this survey were found in (a) the IEEE Xplore digital library, (b) the Google scholar platform, (c) the online Elsevier platform, and d) the online MDPI platform.

Keywords utilized were: “Coverage Path Planning”, “Decomposition methods”, “CPP methods”, “Multi-robot CPP methods”, “Cell decomposition”, “Unmanned Aerial Vehicles”, “Energy optimal path”, “Energy-aware approaches”, “Multi-robot systems”, “Robot coverage”, “Robot kinematics”, and “UAV Remote Sensing”. Initially, the resulting papers (approximately 170) were filtered by choosing the ones referring to CPP algorithms, decomposition methods, multi-robots and multi-UAV coverage path strategies, and energy-awareness CPP algorithms.

The 170 aforementioned publications reviewed for the decomposition methods, single or multi-robot CPP strategies, multi-UAV CPP methods, and UAV energy-saving algorithms. From the 170 papers, 128 were classified according to the relevance of the survey’s scope and their overlapping information. In the end, 128 papers were analyzed for their approaches and their correlation to categorize in sub-sections of decomposition methods, CPP methods, and energy-saving algorithm, of which 88 made it into the refined version of the present survey.

## 3. Results

Choset [19] classified the CPP algorithms according to the decomposition used. Most CPP algorithms decompose the area of interest in cells. This method is preferable for irregular areas. On the other hand, when the area of interest is a regular shape, it does not require any decomposition for single coverage of UAV. Table 2 at the end of this section summarizes the decomposition methods and presents the CPP approach, the decomposition method, the algorithm processing, the shape of the area of interest, and the corresponding reference.

### 3.1. No Decomposition

**Figure 1:**
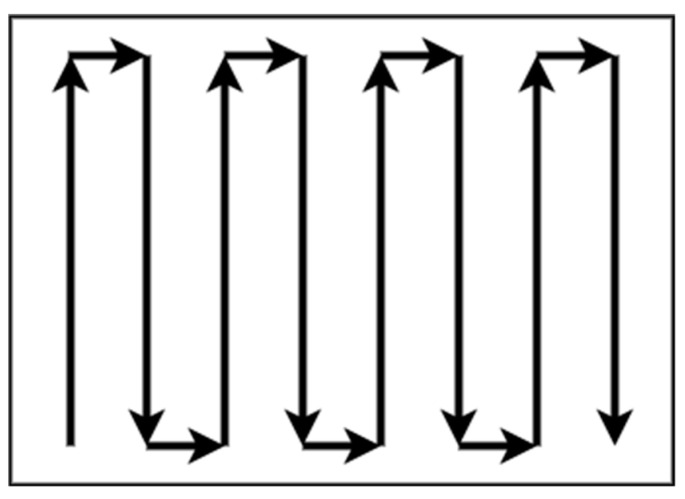
Boustrophedon pattern.

There is no need for decomposition in areas with regular shapes and without complexity, such as rectangular areas. Patterns with simple path planning, such as boustrophedon or square, are adequate for total coverage of a non-complex area without overlapping. The boustrophedon method, which means “the way of the ox,” is a pattern of simple back and forth motion along the longest side of the polygon, as shown in Figure 1 [33,34,35].

The literature assumes that the actual path is closely true to the plan when this method is executed from a ground vehicle. On the other hand, UAVs are aerodynamically directly affected by the direction and intensity of the wind, which means that the actual trajectory of the flight in most cases is not close to that planned.

**Figure 2:**
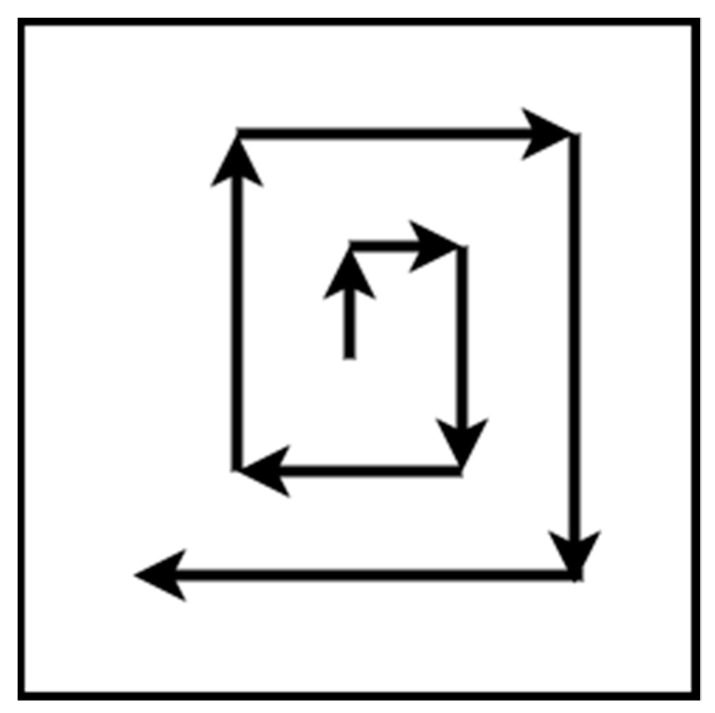
Square pattern.

The square method is represented by Andersen [36], and it is a pattern for a search and rescue mission. The flight path is straight lines with right 90 degrees turns. The pattern starts from the center of the area of interest and expands until the borders, as shown in Figure 2.

### 3.2. Exact Cellular Decomposition

The cellular decomposition methods are based on dividing an irregular space into cells. One class of these methods is the exact one. The exact cellular decomposition method decomposes the irregular space into cells, and their connections produce an accurate free space composition. Accurate methods are complete because they guarantee the finding of an accessible path, if any [37]. The sub-areas that arise from the decomposition can be covered from a single UAV or multiple UAVs. There are patterns for a single UAV, such as boustrophedon and spiral for polygon and concave areas. Nevertheless, there are strategies for the cooperation of multiple UAVs in order to minimize the coverage time [38].

### 3.3. Trapezoidal Decomposition

**Figure 3:**
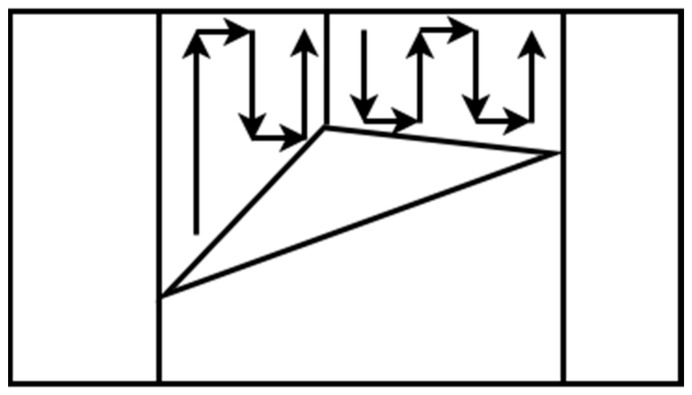
Trapezoidal decomposition.

One exact cellular decomposition technique for irregular spaces that can give a complete coverage path is trapezoidal decomposition. This method is classified in the offline category of algorithms because it does not use remote-sensing information [39,40]. Each cell is a trapezoid in this method, and simple methods such as back and forth can be used to cover every cell. The coverage can be achieved by an exhaustive walk that generates a path to cover each cell to execute the path using back and forth motions such as boustrophedon, as shown in Figure 3. Often, this method is used for agriculture applications where the fields are polygonal and clear from obstacles. Oksanen and Visala [41] introduced an algorithm for CPP in agricultural fields and used the path cost function to optimize the final path.

### 3.4. Boustrophedon Decomposition

**Figure 4:**
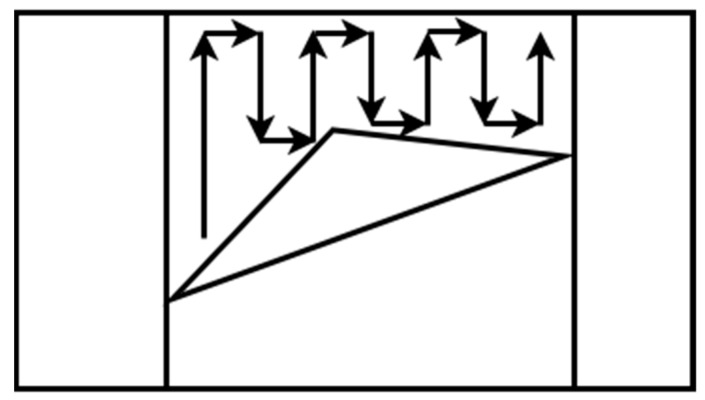
Boustrophedon decomposition.

Trapezoidal decomposition produces many cells, some of which can be merged. This characteristic is a disadvantage because as many cells exist, the coverage path will be longer. To overcome this limitation, a method that creates nonconvex cells is needed. The boustrophedon cellular decomposition is similar to trapezoidal decomposition but considers vertices in the area called critical points [33,35]. The boustrophedon decomposition reduces the number of cells compared with trapezoidal decomposition, which means shorter path planning, as shown in Figure 4. As the trapezoidal decomposition, this method is for polygonal areas, and the environment of the coverage area should be known. For this reason, it can be classified as an offline method.

### 3.5. Morse-Based Decomposition

**Figure 5:**
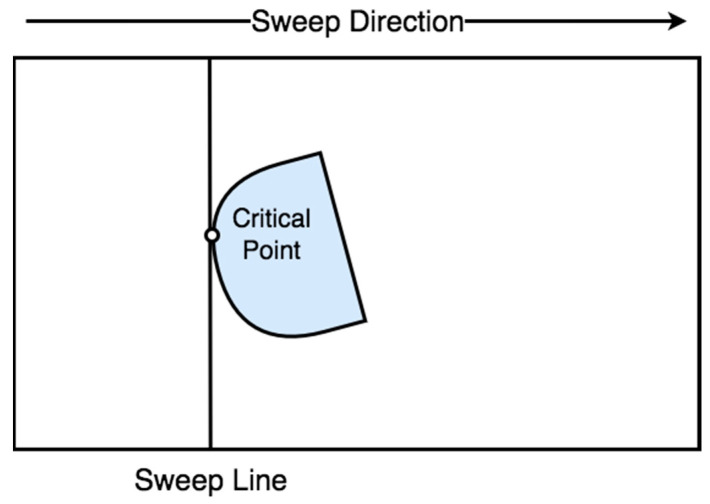
Morse-based decomposition.

Another cellular decomposition method proposed by Acar et al. [42] is based on Morse functions [43]. The Morse-based decomposition method has the advantage of different cells shapes such as circular and can be applied in any dimensional space, such as concave, polygon, and irregular space. The cell decomposition is succeeded with a slice that sweeps through the area of interest. A slice is discontinued at the critical point of the Morse function, which is restricted from the obstacle boundaries, as shown in Figure 5. This method uses information concerning the area during motion planning. For this reason, the method can be classified as online [44,45].

### 3.6. Online Topological Coverage Algorithm

**Figure 6:**
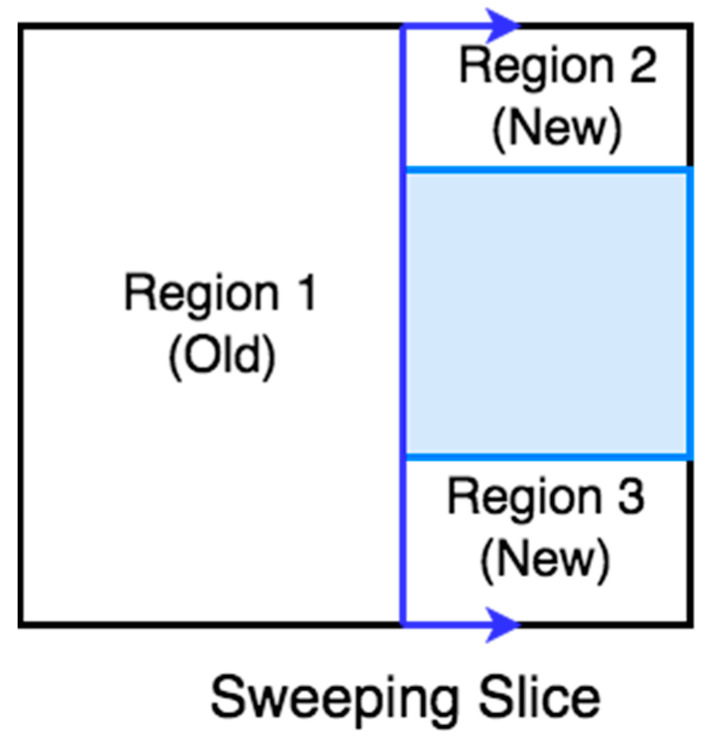
Slice decomposition.

Wong [46] presented an algorithm that finds the cell boundaries online using slice decomposition. Slice decomposition is a method for determining the cell boundaries using a sweeping line over the area of interest. As the line sweeps over the area, it separates the obstacles and free space in two regions or more, as shown in Figure 6. The algorithm constructs a topological map using the slice decomposition on the area of interest [47].

### 3.7. Contact Sensor-Based Coverage of Rectilinear Environments

Butler et al. [48] present an exact cell decomposition algorithm for contact sensor-based robots for online coverage of the rectilinear environment. In contact sensor-based coverage, the robot’s path is cycling with retracing, while at the same time it repeatedly constructs a cellular decomposition of the area of interest. When a robot’s full-cycle path is unsuccessful, it chooses a new path based on its position and environment. The robot’s motion depends on the area’s cell decomposition state, updated as the CPP progresses.

### 3.8. Grid-Based Methods

**Figure 7:**
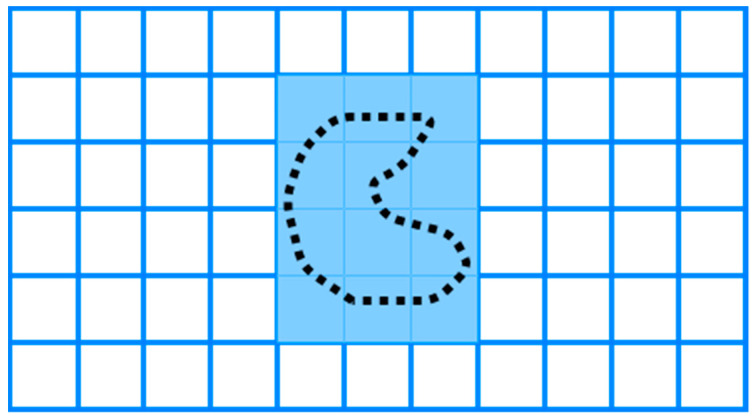
Grid-based decomposition.

Grid-based methods are classified as approximate cellular decomposition due to the restriction of the grid’s shape, which is uniform in space. It is impossible to represent precisely the shape of the target space and its obstacles [19]. The grid-based methods decomposed the space into uniform grid cells, which can be squares or other shapes, as shown in Figure 7. Moravec and Elfes [49] proposed a grid map presentation based on a sonar mounted on a mobile robot mapping an indoor environment.

#### 3.8.1. Wavefront Algorithm

The first CPP’s grid-based method was proposed by Zelinsky et al. [50]. Their method has a start cell and a goal cell. A grid represents the coverage area, and a wavefront algorithm is used from the goal cell to the start cell. Its operation is based on propagating a “wavefront” from the target cell passing through the free cells and bypassing all obstacles to the starting cell.

**Figure 8:**
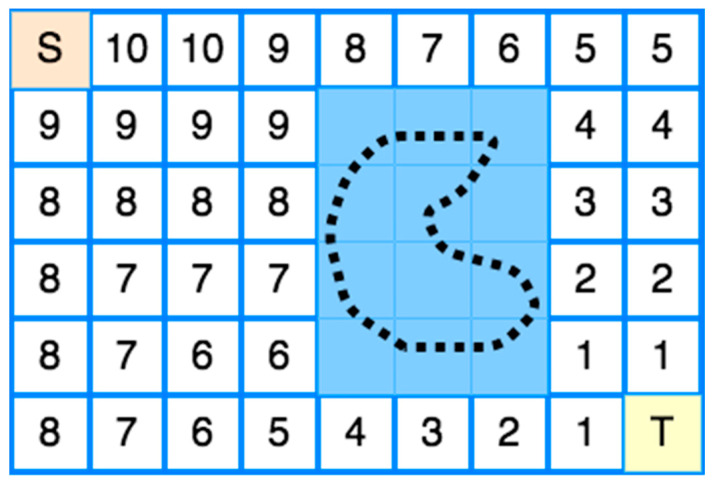
Wavefront Transmission from starting cell (S) to target cell (T).

More specifically, the transmission of the “wavefront” from the target cell to the starting cell is used to assign specific numbers to each cell of the grid, as shown in Figure 8. Firstly, 0 is assigned to the target cell and then 1 to all adjacent cells. Then, all the other adjacent cells of 1 to which no number has been assigned are assigned 2. The process repeats incrementally until the wavefront reaches the starting point [13,20]. The environment should be known in this method, so the method can be classified offline.

Nevertheless, Shivashankar et al. [51] proposed a wavefront algorithm to accomplish an online CPP with a mobile robot in an unknown spatial environment.

#### 3.8.2. Spanning Tree Coverage

**Figure 9:**
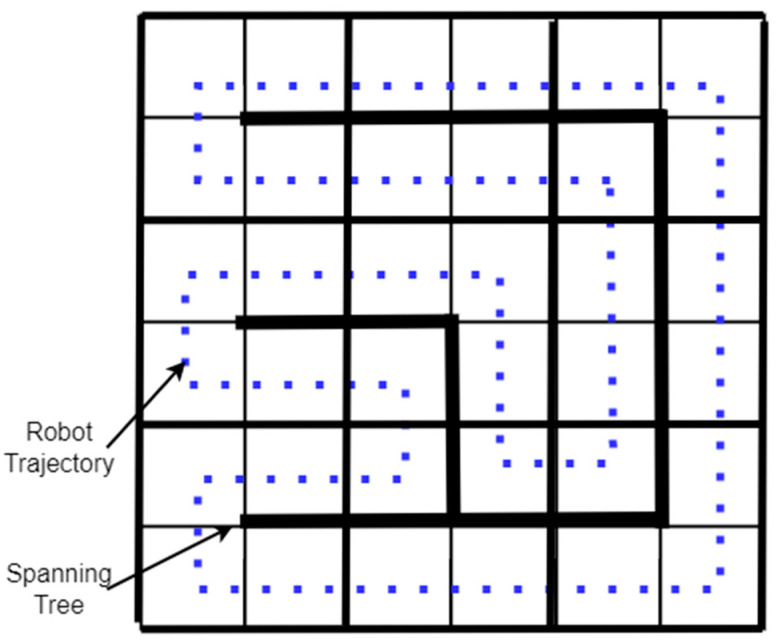
Spanning Tree-based coverage.

The spanning tree coverage (STC) algorithm solves the problem of covering an area using a robot [38]. The method used by the STC algorithm is first to decompose the region into cells and calculate a connecting tree of the resulting graph. Finally, the robot’s path starts near the “connecting tree” and follows its perimeter, as shown in Figure 9 [37]. A Spiral-STC algorithm was proposed by Gabriely and Rimon [52]. This online method converts the space into a grid map. The mobile robots execute a spanning tree-generated spiral path using onboard sensors.

### 3.9. Neural Network-Based Coverage on Grid Maps

**Figure 10:**
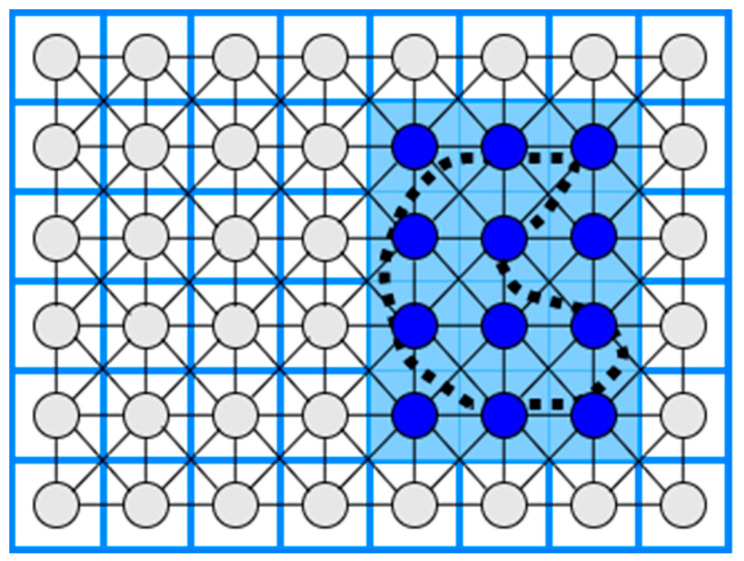
Neural Network-based coverage.

The CPP using a neural network is an online coverage method. First, in a 2D coverage area, a grid map is constructed where the length of the diagonal of each cell is equal to the coverage radius of the robot (e.g., the coverage radius of a robotic broom), and then a neuron is associated with each cell in the grid. Each neuron is connected to the eight primary neighboring neurons, as shown in Figure 10. Finally, the robot’s path to the coverage area is executed by knowing each output value of each neuron at a given time, so that the robot is attracted to cells it has not visited while at the same time being rejected by cells it has visited [53,54].

**Table 2:** CPP and decomposition methods.

CPP Approach	Decomposition Method	Algorithm Processing	Shape of Area	Reference
Boustrophedon	None	Offline	Rectangular	[33,34,35]
Square	None	Offline	Square	[36]
Boustrophedon, Spiral	Exact cellular	Offline	Polygon, Concave	[37]
Back and Forth	Trapezoidal	Offline	Polygon	[39,40]
Boustrophedon	Boustrophedon	Offline	Polygon	[33,35]
Boustrophedon	Morse-based	Online	Any dimensional	[42]
Online Topological	Slice	Online	Polygon	[46]
Contact Sensor-based	Exact cellular	Online	Rectilinear	[48]
Wavefront	Approximate cellular	Offline	Polygon, Concave	[50]
Wavefront	Approximate cellular	Online	Polygon, Concave	[51]
STC	Approximate cellular	Offline	Polygon, Concave	[37]
Spiral-STC	Approximate cellular	Online	Polygon, Concave	[52]
Neural Network-based	Approximate cellular	Online	Polygon, Concave	[53,54]

### 3.10. Multi-Robot CPP Strategies

Multiple robots have an advantage over single robotic systems [24]. The use of multiple robots accelerates coverage of an area of interest. The problem of covering an area with multiple robots lies in the calculation of optimal routes in order to minimize the coverage time [38]. Using multiple robots in a CPP work reduces the completion time due to workload division [20]. This section discusses multi-robot coverage methods based on single robot approaches, multi-robot strategies, and multiple UAVs to cover an area of interest. Some drawbacks of multiple UAV strategies are spatial orientation and communication difficulties. Table 3 at the end of this section summarizes the multi-robot CPP strategies and presents the CPP approach, the decomposition method, the algorithm processing, and the corresponding reference.

### 3.11. Multi-Robot Boustrophedon Decomposition

Rekleitis et al. [16] presented a set of online algorithms for solving the CPP using a group of mobile robots in an unknown environment. The algorithms employ the same planar cellular decomposition as the Boustrophedon single robot coverage algorithm, with additions to manage how robots cover a single cell and distribute among cells. Their solution takes into account the team members’ communication limitations. The robots serve two roles to accomplish coverage where some members, known as explorers, cover the boundaries of the actual target cell, while others, known as coverers, conduct basic back-and-forth motions to cover the cell.

### 3.12. Multi-Robot Spanning Tree Coverage

Their experimental data reveal that their technique outperforms multi-robot spanning tree coverage (MSTC) by a significant margin. Nevertheless, the coverage time of an area with the multi-robot forest coverage (MFC) algorithm is shorter than the MSTC algorithm [38]. Moreover, an online, robust version of MSTC was provided by Hazon et al. [55]. They show that the approach is robust analytically, providing as much coverage as a single robot can.

### 3.13. Multi-Robot Neural Network-Based Coverage

A neural network approach for multi-robot coverage where each robot sees all the others as obstacles and the avoidance ability of stalemate situations was proposed by Luo and Yang [54,56,57]. The multi-robot neural-network based coverage is inspired by single robot neural-network coverage. During the coverage of the irregular-shaped area of interest, the robots see each other as moving obstacles.

### 3.14. Multi-Robot Graph-Based and Boundary Coverage

Easton and Burdick [58] presented a two-dimensional boundary coverage method for multiple robots. A team of robots must inspect all points on the boundary of the two-dimensional target environment, and each robot’s inspection routes are planned to use a heuristic search. The planned paths cover the entire boundary. Moreover, the algorithm has been validated by simulations. The multi-robot boundary coverage is inspired by the need to inspect the blade surfaces inside a turbine.

**Table 3:** Multi-robot CPP strategies.

CPP Approach	Decomposition Method	Algorithm Processing	Reference
Boustrophedon	Exact cellular	Online	[16]
Spanning Tree Coverage	Approximate cellular	Online	[55]
Neural network-based	Approximate cellular	Online	[54,56,57]
Graph-based and Boundary	Approximate cellular	Offline	[58]

### 3.15. Multi-UAV CPP Methods

The number of applications where UAVs can be used is increasing as remote-sensing technology is developed. In the literature, there are a lot of multi-UAV CPP methods using different coverage algorithms with heterogeneous or homogeneous UAVs that were used in a variety of applications, such as agriculture [59], surveillance [60], mapping [61], and search and rescue missions [62]. Table 4 at the end of this section summarizes the multi-UAV CPP strategies and presents the CPP approach, the type of UAVs, the algorithm processing, the evaluation metrics, and the corresponding reference.

### 3.16. Multi-UAV Coverage

In the agricultural sector, Barrientos et al. [13] proposed a method for area coverage using a fleet of mini aerial robots. Their method divides the area of interest in k non-overlapping subtasks and assigns them in k UAVs. A decentralized method for surveillance missions using homogeneous UAVs was proposed by Acevedo et al. [63]. This method’s primary goal is to minimize latency, which means a short sharing time of information between the UAVs. In a later work, Acevedo et al. [64] developed a method for surveillance in urban environments with heterogeneous UAVs that fly at low altitudes and avoid obstacles. Finally, in their most recent work, Acevedo et al. [65] developed a method based on grid-shape area partition, which can readjust the area shape and UAVs’ capacity.

A terrain coverage method using a fleet of heterogeneous UAVs was presented by Maza and Ollero [61]. Their method divides the irregular-shaped area of interest per each UAV capability, such as total flight time. Each partition is assigned to a UAV that plans a zig-zag covering pattern according to the area’s characteristics to minimize the number of turns. The method was validated in simulation.

A coverage algorithm for fixed-wing UAVs with the ability for obstacle and previously scanned regions avoidance was presented by Xu et al. [23,66]. Their method uses boustrophedon cellular decomposition [33], an exact cellular decomposition, and presents better accuracy than trapezoidal decomposition. The method can be classified as online in the phase of region scanning and offline in the coverage phase.

### 3.17. Back-and-Forth

Maza and Ollero [61] present a cooperative technique using heterogeneous UAVs in a convex polygonal area. A ground control station divides the area into sub-regions and assigns them to every UAV by the capability and starting position. Every UAV calculates back-and-forth patterns according to the camera footprint to reduce the number of turns.

### 3.18. Spiral

Balampanis et al. [67,68] present a spiral CPP algorithm using multiple heterogeneous UAVs. The area of interest is divided according to UAVs sensing capabilities using a constrained Delaunay triangulation (CDT) [69]. The CDT generates triangle cells that match almost exactly the shape of the area of interest. To make the triangles more uniform, they applied Lloyd optimization [70]. Then, a spiral algorithm generates the coverage pattern for each sub-area. This method can generate smoother trajectories considering avoiding no-fly zones and the shape of the coverage area. However, it generates more extensive coverage paths and a higher number of turns than classical grid decomposition and motion methods [71,72].

### 3.19. Multi-Objective Path Planning (MOPP) with Genetic Algorithm (GA)

Hayat et al. [73] propose multi-objective path planning (MOPP) with a genetic algorithm (GA) for search and rescue missions using multiple UAVs. The mission is divided into two phases: search and response. The search phase monitors an event to guarantee the total coverage in a given area, and the response phase spreads detection updates on the network. The MOPP algorithm performs the planning task during the search, while the GA minimizes the mission completion time. As a result, the method can be classified as offline in the search phase and online in the response phase.

### 3.20. Genetic Algorithm (GA) with Flood Fill Algorithm

Based on the Trujillo et al. [74] approach, Darrah et al. [75] present a CPP method for missions over more extensive areas using multi-UAVs. The method produces equitable sub-areas of the area of interest to cover by multi-UAVs or several flights performed by a single UAV. The flood fill algorithm integrated with game theory was applied to partition the area of interest. Each UAV is a player and has a starting position. According to a predefined pattern in a diamond shape, the UAVs take turns flooding the neighbor cells. The UAVs cannot fly over building cells or cells previously occupied by other UAVs. The partitioning method guarantees an approximate amount of work for each assigned UAV by balancing the tasks. An improved version of the approach proposed by Trujillo et al. [74] was used for each sub-area’s coverage trajectories. The method can be classified initially as offline and then as online.

**Table 4:** Multi-UAV CPP strategies.

CPP Approach	Type of UAVs	Algorithm Processing	Evaluation Metrics	Reference
Sub-perimeter method	Homogeneous	Online	Minimize latency	[63]
Back-and-Forth	Homogeneous	Online/Offline	Total path length Time coverage	[23,66]
Back-and-Forth	Heterogeneous	Offline	Number of turns	[61]
Spiral	Heterogeneous	Offline	Coverage path, Number of turns	[67,68]
Multi-Objective Path Planning with GA	Homogeneous	Offline/Online	Mission Completion Time	[73]
GA with flood fill algorithm	Homogeneous	Offline/Online	Path length	[74,75]

### 3.21. Energy-Saving CPP Algorithms

In the literature, there are a lot of CPP strategies for energy saving. One method for energy saving proposed by Lawrance and Sukkarieh [25] is the energy exploitation of the wind using a small gliding UAV. The authors present an algorithm that generates energy gain paths according to the UAV’s constraints, the field’s wind conditions, and static and dynamic soaring. One of the limitations of this method is the requirement for prior knowledge of the field’s wind conditions. In future research, an online stochastic wind estimation and planning method using current wind conditions of the field should be developed.

Another method for minimizing the power consumption of a UAV is reducing the number of turns of the CPP. Torres et al. [76] present an algorithm that reduces the number of turns and the total flying path to minimize battery consumption.

The effect of wind direction and intensity on the time of mission completion was presented by Coombes et al. [77]. The authors used a fixed-wing UAV and the boustrophedon method to cover the area of interest. Their simulated experiments used a constant direction of the wind and six different speeds, and for the coverage path used different directions of the fixed-wing UAV motion from 0 to 360 degrees in increments of 10 degrees. The results showed that the direction of the coverage path should be 90 degrees to the wind direction to minimize the coverage time. Furthermore, the direction of the turns is directly affected by the vertical component of the wind. In a later work, Coombes et al. [78] presented the flight time in wind (FTIW) function, which computes the total flight time for a total coverage of the area of interest. The flight time needed for the total coverage of the area is less than the previous methods. Their approach was validated after simulations and real flights.

An energy-efficient back and forth CPP algorithm proposed by Di Franco and Buttazzo [18] computes the best motion trajectory and the maximum altitude according to the ground sample distance (image resolution) to minimize the number of turns. Another approach for energy efficiency is to find an optimal constant speed according to the coverage path. An energy-aware spiral CPP algorithm uses wider angle turns to minimize the acceleration and deceleration to maintain an optimal constant speed Cabreira et al. [79]. After simulated and real flights, the most energy-efficient CPP method between energy-efficient back and forth CPP [18] and the energy-aware spiral CPP approach proposed by Cabreira et al. [79] for a convex area was the energy-aware spiral CPP method which adopted the energy model proposed by Di Franco and Buttazo [80].

Another energy-aware CPP algorithm for UAVs was proposed by Li et al. [81], where the algorithm has three stages. In the first stage, the algorithm builds a 3D terrain model. In the second stage, constant power consumption is computed by total take-off weight, flight speed, and air friction. In the third stage, a genetic algorithm generates an energy-optimal coverage path, which represents the amount of energy consumption in every part of the path.

Another problem concerning UAV energy consumption is the deceleration and acceleration at every turn of a conventional trajectory such as boustrophedon. Artemenko et al. [82] present an algorithm that modifies conventional trajectories using Bézier curves, smoothing the turns on a given path to minimize deceleration and acceleration before and after the turning point. The authors concluded that their algorithm could reduce energy spending compared to conventional algorithms. Restrictions, such as the UAV motion and camera’s location, can be overcome using integer linear programming. Ahmadzadeh et al. [83] present a cooperative coverage technique with critical time for rectangular areas utilizing several fixed-wing heterogeneous UAVs, some carrying a frontal camera, flying circular paths and some of them carrying a camera on the left side, flying straight lines with left turn paths. Their proposed method uses four fixed-wing UAVs covering 100% of the area of interest instead of the simple methods covering 80%. The proposal was validated in simulation tests and real flights.

Araujo et al. [84] propose an algorithm where the workspace is divided into sub-areas assigned to each UAV according to its relative capability. According to the kinematics constraints of the UAVs, the algorithm generates an optimal number of stripes to minimize the number of stripes and eventually the number of turns, which means less energy consumption.

Majeed and Lee [85] present a CPP method for UAV low-altitude navigation in three-dimensional urban areas with fixed convex obstacles based on footprint sweeps fitting and a sparse waypoint graph. The primary goals of the proposed approach are to reduce computational time, the number of turns, and path overlapping while minimizing the total coverage path of the area of interest. The suggested method outperforms the similarly related CPP approaches according to simulation findings.

In a later work, Majeed and Hwang [86] present a CPP algorithm for UAV navigation to cover areas of interest (AOIs) surrounded by obstacles in three-dimensional urban areas with fixed obstacles. The proposed method is applicable in a wide range of practical applications that involve computing a low-cost coverage for spatially distributed AOIs in an urban environment. However, the proposed algorithm has not incorporated and tested for constraints and limitations, such as image resolution and UAV battery.

Cheng et al. [87] present a bio-inspired method for cooperative coverage. This method represents the trajectory of each UAV as the B-spline curve containing control points. This optimization problem aims to maximize the desirability of a path by combining four variables: path distance, minimum turning angle, maximum pitch rate, and superposition of the actual trajectory over different UAV trajectories. According to the authors, the beginning and last control points are at the area’s borders because the UAV always travels from left to right. The ant colony optimization (ACO) algorithm was adapted for coverage with multiple UAVs by Kuiper and Nadjm-Tehrani [88]. The *y*-axis in the intermediate control points is optimized using the ACO algorithm to maximize the coverage. Several ants are launched during the algorithm repetitions, passing through the starting, intermediate, and endpoints.

Table 5 summarizes the energy-saving CPP methods reviewed in this paper according to the method used for energy saving. The table presents the CPP method, the energy-saving factor, the type of UAV, and the corresponding reference.

## 4. Discussion

The CPP problem using UAVs in areas of interest with different shapes and environmental conditions has been studied by several authors. Standard-shaped areas of interest, such as polygons and rectangles, do not require decomposition and can be covered by boustrophedon and spiral patterns. Generally, no decomposition methods, such as back-and-forth, require low computational cost to find the path trajectory. The main issue of these patterns is not considering that the UAVs are directly aerodynamically affected by the environmental conditions, which means the actual trajectory of the flight in most cases is not close to that planned.

In more complex and irregular areas of interest, a cellular decomposition method may be applied to split the area of interest into subregions. The subregions can be covered by different CPP methods to obtain the optimum path to minimize the total path and the total coverage flight time. Multi-UAV cooperative strategies are also being studied using the decomposition method according to the capabilities of the UAVs.

When the vehicle used for the proposed CPP algorithms is a UAV, there is the limitation of the motion constraints, such as the feasible trajectory of fixed-wing UAVs. However, the CPP methods plan the coverage path according to a performance metric. These approaches do not consider the UAVs’ environmental factors and aerodynamic and flight limitations.

A further study is necessary for the area of CPP methods using UAVs. The coverage algorithms should consider the constraints of the aerial vehicles, such as the actual path trajectory rather than that planned. Moreover, the environmental factors in the area of interest that affect the path, the time, and the actual flight path should also be considered. According to all these mutable factors, an offline CPP method will not achieve optimal path planning, but an online CPP method considering all these factors and re-planning the trajectory will achieve the optimal coverage path within minimum time.

In recent years, many new CPP algorithms have been developed for energy-efficiency and awareness. The approach using a glider UAV for soaring limits early knowledge of the field’s wind conditions. Otherwise, the method is less effective in a situation where the knowledge of wind conditions is limited [25]. In approaches where engine-driven UAVs are used, there are some methods or combinations for energy saving. A method for power saving in non-complex areas is reducing the number of turns in the UAV’s trajectory to minimize the total path and the acceleration’s power consumption after every turn, and eventually the total coverage time of the area of interest [20,76]. In approaches for energy saving, considering the direction and intensity of the wind was validated as the UAV’s path should be vertical in the wind direction, and the turning maneuvers against the wind direction [77,78]. This approach can be combined with the previous method for greater energy saving.

Two more approaches that can be used in combination with the previous methods for further energy saving include minimizing the UAV’s turns according to the GSD [18]. A spiral CPP algorithm uses wider angle turns to maintain a constant speed [67] or an algorithm for a conventional trajectory that modifies the turns for smoother motion [70] to minimize the deceleration and acceleration before and after the turning point. Another energy-aware algorithm computes the take-off weight, flight speed, and air friction to generate an energy-optimal path [81].

In convex areas, there are approaches using multiple UAVs to divide into sub-areas and assign each sub-area according to the UAV’s capability, such as motion, sensors onboard, and total endurance flight time [83,84].

The proposed energy-efficient UAV CPP methods aim to minimize the total flight time and the coverage path length to save energy. However, the performance metrics are based on the path trajectory without considering other constraints, such as UAV aerodynamics and environmental conditions. For example, in a convex area, a CPP method with a performance metric for minimum path trajectory may produce very sharp turns. Meanwhile, it is infeasible for a fixed-wing UAV to obtain the planned trajectory due to its aerodynamics constraints. Another variable affecting the UAV’s actual trajectory is the wind’s direction and intensity. The UAV will consume more energy than a more extensive path length with smoother turns considering all these limitations.

A further study is necessary to combine all of the above constraints to develop new energy-efficient UAV CPP methods that consider variables, such as the vehicle kinematics and environmental conditions offline and online. A research direction to develop UAV CPP methods to maximize energy-saving should combine machine learning or deep learning and IoT onboard sensors in order to develop a CPP approach that will plan offline and adapt online the coverage path trajectory according to the main performance metrics, such as UAV kinematics constraints, and the information retrieved from onboard sensors such as wind conditions.

## 5. Conclusions

This paper presented a survey of coverage path planning according to the decomposition methods, such as no decomposition, exact, and approximate decomposition methods. Different shapes of the area of interest, such as concave, rectangular, and polygon, are considered in this survey. We focused on simple path planning patterns, such as boustrophedon and spiral, and more complex approaches such as grid-based methods. We also presented multi-robot and multi-UAV CPP strategies that aim to accelerate the coverage area by focusing on optimal routes.

Some authors in more complex missions and areas use multiple UAVs to overcome their endurance limitations. However, this approach demands computational complexity to solve communication issues and coordinate the UAVs. The coordination of the UAVs requires a ground control station, which presents many communication failures in real-world scenarios.

CPP methods with simple path planning, such as boustrophedon [33] and square [36], are preferred over cellular decomposition methods for regular shapes without complexity. These CPP methods need less computational time, but they have limitations when UAVs use them. Exact cellular decomposition CPP methods are preferable in more complex area shapes, such as a polygon or concave. The boustrophedon cellular decomposition [37] is similar but better than trapezoidal decomposition [39,40] when the shape of the area has many vertices. The boustrophedon overcomes the trapezoidal decomposition by reducing the number of cells, which means shorter path planning. The morse-based decomposition [42] has the advantage over the other decomposition approaches in that it can produce different cell shapes such as circular and can be applied in any dimensional space. The contact sensor-based coverage is preferable in a rectilinear environment and for online coverage of the area because the coverage trajectory is updated as the CPP progresses.

Furthermore, we present UAVs’ energy-saving CPP algorithms, which enhance the energy efficiency using optimal coverage methods and approaches, such as the sub-area assignment of the area of interest according to the capability of the UAV in a multi-UAV CPP strategy.

Finally, several kinds of research have been performed for UAV energy-aware methods in the literature. However, a remaining issue for further research is the combination of these techniques with machine learning, deep learning, and IoT sensors to develop a new, dynamic CPP method that will maximize energy-saving compared to the proposed energy-efficient CPP methods.

## Figures and Tables

**Table 1:** Related surveys.

Related Work	Decomposition Methods	Multi-Robot Strategies	UAV CPP Methods	Multi-UAV CPP Methods	Energy-Saving Algorithms	Comparison of Energy-Saving CPP Methods
Cabreira et al. [30]	✓	✕	✓	✓	✓	✕
Galceran and Carreras [20]	✓	✓	✕	✕	✕	✕
Almandhoun et al. [31]	✓	✓	✕	✓	✕	✕
Chen et al. [32]	✕	✕	✓	✓	✕	✕
Our work	✓	✓	✓	✓	✓	✓

**Table 5:** CPP energy-aware methods.

CPP Method	Energy-Saving Approach	Type of UAV	Reference
Energy gain path	Energy exploitation of the wind	Fixed-wing	[25]
Back and Forth	Reducing the number of turns and the total flying path	Rotorcraft	[76]
Boustrophedon	The direction of the UAV path and the turns according to the wind direction	Fixed-wing	[77]
Back and Forth	Altitude maximization according to the Ground Sample Distance to reduce the number of turns	Rotorcraft	[18]
Spiral	Wider angle turns to minimize the acceleration and deceleration	Rotorcraft	[79]
Three stages energy optimal path	An energy-aware algorithm computes the take-off weight, flight speed, and air friction to generate an energy-optimal path	Rotorcraft	[81]
Smoothing turns	Smoothing the turns on a given path to minimize deceleration and acceleration before and after the turning point	Rotorcraft/Fixed-wing	[82]
Circular and straight lines with left turns paths	Cooperative coverage algorithm with critical time	Multiple Fixed-wing	[83]
Back and Forth	Minimizing the number of stripes and eventually the number of turns	Multiple Fixed-wing	[84]
Back and Forth	Reduce computational time, the number of turns, and path overlapping while minimizing the total coverage path	Rotorcraft	[85]
Back and Forth	Reducing the computational time and path length for the inter-regional path, the number of turning maneuvers, and path overlapping	Rotorcraft	[86]
ACO with Gaussian distribution functions	Path length, rotation angle and area overlapping rate	Rotorcraft/Fixed-wing	[87]
